# Supplementary material for: Low knowledge about hepatitis B prevention among pregnant women in Kinshasa, Democratic Republic of Congo
Source: PLOS Glob Public Health. 2022 Sep 23;2(9):e0000450. doi: 10.1371/journal.pgph.0000450 (PMC10021814; doi:10.1371/journal.pgph.0000450)
Supplement: S1 Questionnaire — This questionnaire was used upon enrollment of both the AVERT and BDI trials. Denoted “incorrect” or “correct” in italics for the purposes of this manuscript (not included in the original knowledge questionnaire). (DOCX) [file pgph.0000450.s001.docx]

Low knowledge about hepatitis B prevention among pregnant women in Kinshasa, Democratic Republic of Congo

**SUPPLEMENT**

| **Read: *Now I am going to ask you some questions about your understanding of Hepatitis B and the risk of passing it on to your baby during pregnancy or childbirth.*** | |
| --- | --- |
| **1.** **In your opinion, how serious a disease is hepatitis B?**  Very serious  Somewhat serious  Not very serious  Not at all serious  Don’t Know  Refuse to answer | **2.** **What are the signs and symptoms of hepatitis B (check all that apply)?**  No symptoms  *CORRECT*    Jaundice (yellowing of skin)  *CORRECT*  Abdominal pain  *CORRECT*    Rash  *CORRECT*    Don’t Know  Refuse to answer |
| **3.** **How can a person get hepatitis B (check all that apply)?**  Through handshakes  *INCORRECT*    Through contact with blood from an infected person  *CORRECT*    Through sharing dishes    *INCORRECT*    Mother-to-child transmission  *CORRECT*  Sexual transmission  *CORRECT*  Through breastfeeding  *INCORRECT*  Don’t Know  Refuse to answer | **4.** **How can mother-to-child transmission of hepatitis B be prevented (check all that apply)?**  Infant vaccination at birt *CORRECT*  Antiviral treatment during pregnancy  *CORRECT*    Don’t Know  Refuse to answer |

**S1 Questionnaire.** **Enrollment questionnaire assessing knowledge, attitudes, and beliefs of pregnant women in the DRC.** This questionnaire was used upon enrollment of both the AVERT and BDI trials. Denoted “incorrect” or “correct” in *italics* for the purposes of this manuscript (not included in the original knowledge questionnaire).
