# Supplementary material for: Low knowledge about hepatitis B prevention among pregnant women in Kinshasa, Democratic Republic of Congo
Source: PLOS Glob Public Health. 2022 Sep 23;2(9):e0000450. doi: 10.1371/journal.pgph.0000450 (PMC10021814; doi:10.1371/journal.pgph.0000450)
Supplement: Attachment — Submitted filename: Response to Reviewers_v3.docx [file pgph.0000450.s002.docx]

**Response to Reviewers**

## Journal Requirements:

1. Please provide separate figure files in .tif or .eps format.

For more information about figure files please see our guidelines:

<https://journals.plos.org/globalpublichealth/s/figures>
<https://journals.plos.org/globalpublichealth/s/figures#loc-file-requirements>

**RESPONSE**: We have uploaded the figure files in .tif format.

2. We noticed that you used “data not shown”/"unpublished data" in the manuscript. We do not allow these references, as the PLOSdata access policy requires that all data be either published with the manuscript or made available in a publicly accessible database. Please amend the supplementary material to include the referenced data or remove the references.

**RESPONSE**: Thank you, we have updated this reference accordingly.

3. Your manuscript is missing the following sections: Introduction. Please ensure these are present, and in the correct order, and that any references to subheadings in your main text are correct. An outline of the required sections can be consulted in our submission guidelines here:

<https://journals.plos.org/globalpublichealth/s/submission-guidelines#loc-parts-of-a-submission>

**RESPONSE**: We have included an Introduction section.

4. We have noticed that you have uploaded Supporting Information files, but you have not included a list of legends. Please add a full list of legends for your Supporting Information files after the references list.

**RESPONSE**: We have added a list of supporting information captions.

Comments to the Author:
Please use the space provided to explain your answers to the questions above. You may also include additional comments for the author, including concerns about dual publication, research ethics, or publication ethics. (Please upload your review as an attachment if it exceeds 20,000 characters)

**Reviewer #1:**

Dear authors,
I enjoyed reading your paper on this very important matter in the DRC. Reading the results on the low awareness and knowledge about HBV shows how important this subject matter is for public health prevention strategies. Thank you for addressing this issue and putting it in numbers.

I find the manuscript as it is presented now not suitable for publication. Because of the high importance of the topic, I suggest a major revision of the manuscript. The manuscript has room for textual improvement, more scientific writing, improved structure and flow, use of subheadings. For feedback on each part of the manuscript please see below. I hope this review will help you to improve the manuscript.

**RESPONSE:** Thank you for your comments and suggestions. We appreciate the opportunity to respond to your feedback and to improve our manuscript. We added subheadings and additional detail in response to your suggestions (see below for changes made in response to each point).

Introduction
I find the introduction a bit short where more information could be provided about the HBV status in the DRC, insights on the numbers in DRC, why you want to target this specific group, a problem statement. L45, do you have estimates on the high endemic HBV status of the DRC?

**RESPONSE:** We have added language describing the burden of HBV among children in the DRC and how HBV MTCT serves as a key driver of chronic HBV in children. We further outlined the importance of HBV knowledge and perceptions among pregnant women in endemic regions to ensure adequate buy-in to HBV PMTCT screening and treatment (**Lines 54-60**).

Methods
The method section states that a secondary analysis was conducted. This means that you did not have to collect the data again but rather used the existing data. If this is so, why do you report on the written informed consent and the ethical approval? It was to me, as a peer-reviewer, not very clear on how you did the secondary analysis, what part of the BDI and AVERT study data you used. Could you please rewrite this section and clarify? In L81 you describe a sum of scores, however the total sum, its meaning, cut off values etc are not described. The reader has to know how to interpret the values e.g. is 9 high or low?

**RESPONSE:** We added language describing what part of the AVERT and BDI studies were used for this secondary data analysis (**Lines 132-114**). We reported informed consent and ethical approval for the two studies, because this is required even in secondary data analyses when describing the parent studies. We also added language to the methods section clarifying the knowledge score (**Lines 135-140**).

Results
Could you please restructure the results, use subheadings and also conduct statistical analysis on factors that might be associated with HBV knowledge.

**RESPONSE:** We have added subheadings to the results section as suggested. Regarding statistical testing, we did not conduct formal hypothesis testing to compare factors between participants with no HBV knowledge and any HBV knowledge. Because this is a secondary analysis of data collected from studies whose primary objectives were not to assess factors related to HBV knowledge, there is likely unmeasured confounding that influences which participants have HBV knowledge. For this reason, we were hesitant to conduct statistical testing to compare covariates between these groups without accounting for confounding, as the results could lead to spurious conclusions about these relationships. We have added additional language about these limitations in the Discussion section (**Lines 340-345)**

L93- L96: this information belongs in the methods section.

**RESPONSE:** Thank you, we have moved this language to the methods section only.

L103: you introduce this info: 0 on our 9-point HBV knowledge assessment scale. Could you please address this scale in the methods section?

**RESPONSE:** We have added language about the scale in the methods section (**Lines 134-140**)

L108: women were slightly younger. What does this mean? Was age a significant factor? Could you add the data to the text?

**RESPONSE:** We have added the mean ages of women with and without any HBV knowledge to the results section of the text (**Lines 172-173**). We have also added language addressing this observed difference in the discussion (**Lines 342-345**).

L153 – L154: this information should be in the text

**RESPONSE:** We have included this information in the Follow-Up Knowledge Assessment section in the results (**Lines 245-246**)

Conclusion:
I believe this heading should be changed into discussion. The first paragraph of the discussion section should wrap up the main results of the study prior to discussing these results in the rest of the discussion section. You can add a final paragraph named conclusion. Also, I am missing what your data really means in the context. I like that you have drafted recommendations.

**RESPONSE:** Thank you for these insights. We have changed the heading accordingly and added language to summarize the results of our study at the beginning of our Discussion section (**Lines 267-272**). We summarize our conclusions in our last two paragraphs of this section where we outline the low levels of preliminary HBV knowledge among pregnant women in Kinshasa and the necessity for education initiatives and screening within the prenatal care infrastructure in endemic areas.

L162: I think you mean ‘had lower HBV knowledge at baseline’

**RESPONSE:** Thank you. We have edited the language accordingly.

L169: you write that education was not significantly associated with hbv knowledge. However, I could not retrieve this data in the tables nor in the results section.

**RESPONSE:** We include information about education levels among participants with any or no HBV knowledge in Table 1. We have also added text to the results referencing this part of the table (**Lines 175-176**).

I hope to have informed you sufficiently.
Good luck.

**Reviewer #2:**

The title said “Low knowledge about hepatitis B prevention among pregnant women in the Democratic Republic of the Congo” as my view it could be good to say “To assess the knowledge or to determine the knowledge level…….” Rather than saying Low knowledge. To say a low knowledge, we have to have some measurement to categorize it low or high or other. Another point here in the title that need attention is the scope of the study; it said “ ……among the pregnant women in the DRC” it is better to put the province or county or district of the study rather than generalizing the finding of two health facility to the country level. In clear term the data that the study obtained might not represent the country as whole so it needs some modification.

**RESPONSE:** Thank you for your suggestions about the title. We agree, it is more appropriate to specify Kinshasa Province rather than Democratic Republic of Congo, and we have adjusted the title accordingly. It is our opinion that a title should convey the primary findings of the manuscript, and we observed that most women in our study had no knowledge about Hepatitis B. We are open to other suggestions about how to phrase this if you or the editors have suggestions for an alternative to “low”.

In the abstract part the study is summarized from line 27 to 33, it is better if the major finding of the knowledge status of each question is putted. In addition, adding the key word at the end of abstract is make ease to the reader to find on search engine, thus it is better to consider keyword of the study.

**RESPONSE:** Thank you for your suggestion within the abstract. We have added the major findings of initial HBV knowledge status to each question (**Lines 36-38**). In addition, we have added the key words to our study.

The intention of introducing of HBV magnitude that used in introduction part is slightly differ from the sentence that used in abstract part line 23 and 24…. Infants infected perinatally with hepatitis B (HBV) are at the highest risk …… so it is also better to more elaborate this sentence in introduction part or boldly show it.

**RESPONSE:** We appreciate and agree with your suggestion to elaborate and clarify the risks associated with MTCT and have added language to the introduction to address this. (**Lines 54-60**)

In method part the sample size determination and sampling technique is expected, it is fascinating to mention it.

**RESPONSE:** Given that this is a secondary analysis of collected data, we had a fixed sample size determined by the AVERT and BDI studies.

On other hand in method part the issue of data quality, how the questionnaire or data collection tool was prepared and validated has to stated. But I fail to get it, so it is better to state the brief of it. Another important point here is how the study operationalizes the knowledge measurement. This study has to incorporate and elaborate how some very pertinent variables were operationalized with its reference if any.

**RESPONSE:** Thank you, we have added additional language about determination of the HBV knowledge score to the methods section (**Lines 134-140**).

Under method part of Line 81 the calculation of knowledge was stated, it is good if the study shows us the cutoff point that show the level of knowledge is mentioned.

**RESPONSE:** Thank you, we have added language about the cutoff for HBV knowledge score (**Lines 138–140**)

The data analysis part is need an important point that determine the statistical analysis is significant or not, meanwhile the p value or other measurement that declare the significance level has to mentioned.

**RESPONSE:** We report the p value for significance testing when we compare the average knowledge score at follow-up and at baseline (**Line 246**). We did not conduct formal hypothesis testing to compare factors between participants with no HBV knowledge and any HBV knowledge, because this is a secondary analysis of data collected from studies whose primary objectives were not to assess factors related to HBV knowledge.

In result part the response rate mentioned on line 118 has to move to the top of result narration paragraph. In addition, it is better to move the statement that mentioned under line 119 and 120 to method part.

**RESPONSE:** This is helpful commentary. We have added this language to the Methods and Results sections, respectively (**Lines 135-136** and **Lines 164-165**).

Regarding the table of sociodemographic characteristics as to me it is better to make it the first table of the article, and also the table need the top and bottom border.

**RESPONSE:** Thank you for this suggestion. We have changed the order of the tables and added a border.

The statement that mentioned on line 125 and 126 is not clear for me.

**RESPONSE:** We have edited this text for clarity (**Lines 210-212**)

Question
1. Did the author believe that the title of study is clearly consistent with result of the study?
2. In result part of table 2, the table grouped as Any HBV Knowledge and No HBV Knowledge. What do the No HBV knowledge mean? It needs more clarification under method part.
3. Did the author convinced that all statistical analysis were performed accordingly?
3. In discussion part the result of study is compared in percent with another study finding that also mentioned in percent. How clearly we know whether the finding is higher than of lower that without showing the exact confidence interval of the percent?

**RESPONSE:** We believe we have addressed these points in the above responses, but please let us know if you still have additional concerns.

After all the paper is potentially publishable once the authors revised their manuscript based on the comments given and responded to each questions accordingly.

**Reviewer #3:**

I would like to thank you for giving me the opportunity to review this well-written manuscript and congratulate the team on their excellent work. Comments on this manuscript primarily focuses on the analysis and interpretation of the data.

Overall, it would be helpful to include information on the distribution of the responses (with HBV knowledge or without HBV knowledge) by different characteristics of the study population as a whole. For example, at the Maternity Center in Binza, the percentages of women who had HBV knowledge versus those who had no HBV knowledge were 11.9% (17/143) and 88.1% (126/143), respectively, whereas at Kingasani, the percentages were 69.3% (95/137) and 30.7% (42/137) respectively. Thus, these data clearly indicated that there was a significant geographical variation in knowledge of HBV. Ideally, the team should continue to examine and stratify the geographic factors (variations) that may influence or explain other factors such as wealth, work, and education to ensure that no confounding factors might affect the conclusions of this study. In addition, it appears that the work, wealth quartile factor may also be related to knowledge of HBV to some degree, based on the data.

**RESPONSE:** The reviewer brings up an important point about the differences between knowledge scores among women who sought care at the two maternity centers. Both maternity centers are located in Kinshasa so do not reflect geographic variation, but they do generally serve populations with different sociodemographic status. We have added language about the potential differences in work and wealth, with the caveat that this study was not designed to assess relationships between demographic factors and HBV knowledge (**Lines 252-255**). Nonetheless, these factors will be important to investigate further in larger research studies. We have added an additional paragraph in the discussion addressing the differences in demographic characteristics among the participants with differential HBV knowledge (**Lines 339-356**).

Lastly, regarding the findings of improving knowledge of HBV over the course of the AVERT trial, based on the design of the study, there might be a bias related to "selection bias" in which the participants who agreed to participate in BDI, regardless of the informal education received from the program, had an inherent, unconscious tendency to actively seek knowledge to manage the conditions once they became aware of the conditions. Additional analysis/explanation may be required to distinguish and quantify the efforts between self-contribution factors and external influences from informal education that contribute to the improvement of HVB knowledge.

**RESPONSE:** This is a helpful point. The available covariates in our dataset are insufficient to identify self-contribution factors that might have led AVERT participants to also enroll in the BDI study. We have added language about potential selection bias among participants in both studies to the Discussion section study limitations (**Lines 373-378**).
